# Supplementary material for: Proteomic and metabolomic profiling of extracellular vesicles produced by human gut archaea
Source: Nat Commun. 2025 Jun 3;16:5094. doi: 10.1038/s41467-025-60271-w (PMC12134236; doi:10.1038/s41467-025-60271-w)
Supplement: Supplementary file 1 — Supplementary Information [file 41467_2025_60271_MOESM1_ESM.pdf]

Supplementary Information

Proteomic and metabolomic profiling of extracellular vesicles produced by human gut archaea

Viktoria Weinberger[1], Barbara Darnhofer[2], Himadri B Thapa[3], Polona Mertelj[1], Régis Stentz[4], Emily Jones[4], Gerlinde Grabmann[5], Rokhsareh Mohammadzadeh[1], Tejus Shinde[1], Christina Karner[6], Jennifer Ober[7], Rokas Juodeikis[4], Dominique Pernitsch[8], Kerstin Hingerl[8], Tamara Zurabishvili[1], Christina Kumpitsch[1], Torben Kuehnast[1], Beate Rinner[6], Heimo Strohmaier[7], Dagmar Kolb[8], Kathryn Gotts[9], Thomas Weichhart[10], Thomas Köcher[5], Harald Köfeler[2], Simon R. Carding[4,11], Stefan Schild[3,12,13], Christine Moissl-Eichinger[1,13*]

[1] Diagnostic and Research Institute of Hygiene, Microbiology and Environmental Medicine, Medical University of Graz, Austria

[2] Core Facility Mass Spectrometry, Medical University of Graz, Graz, Austria

[3] Institute of Molecular Biosciences, University of Graz, Graz, Austria

[4] Food, Microbiome and Health Institute Research Programme, Quadram Institute Bioscience, Norwich, United Kingdom

[5] Vienna BioCenter Core Facilities GmbH, Metabolomics, Vienna, Austria

[6] Core Facility Alternative Biomodels & Preclinical Imaging, Medical University of Graz, Graz, Austria

[7] Core Facility Flow Cytometry, Medical University of Graz, Graz, Austria

[8] Core Facility Ultrastructure Analysis, Medical University of Graz, Graz, Austria

[9] Core Science Resources, Quadram Institute Bioscience, Norwich, United Kingdom

[10] Center for Pathobiochemistry and Genetics, Medical University of Vienna, Vienna, Austria

[11] Norwich Medical School, University East Anglia, Norwich, United Kingdom

[12] Field of Excellence Biohealth – University of Graz, Graz, Austria

[13] BioTechMed Graz, Austria

*Corresponding Author. Christine Moissl-Eichinger. christine.moissl-eichinger@medunigraz.at

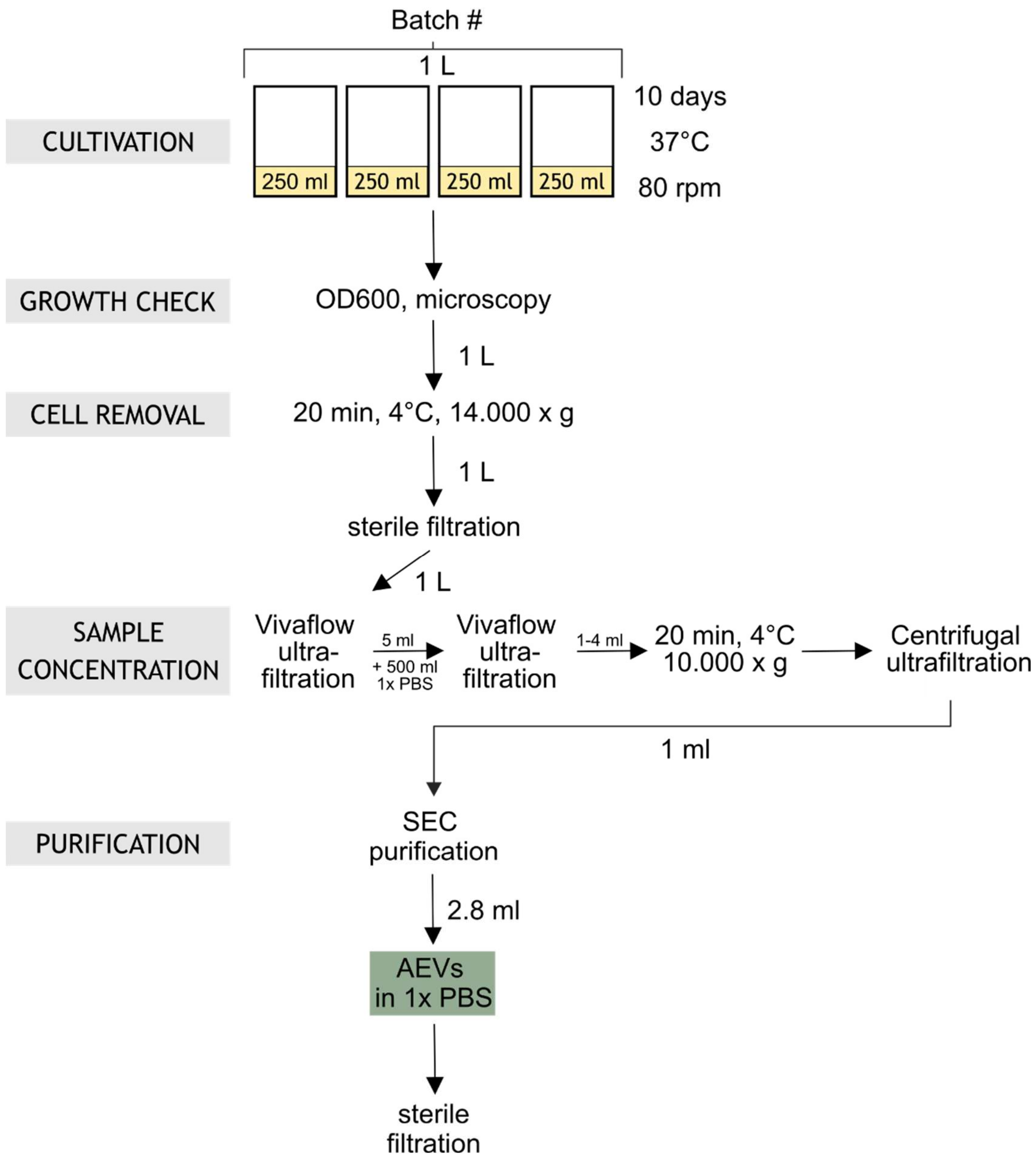

Supplementary Figure S1: Workflow of archaeal extracellular vesicle (AEV) preparation and isolation. Abbreviations: (SEC) Size exclusion chromatography.

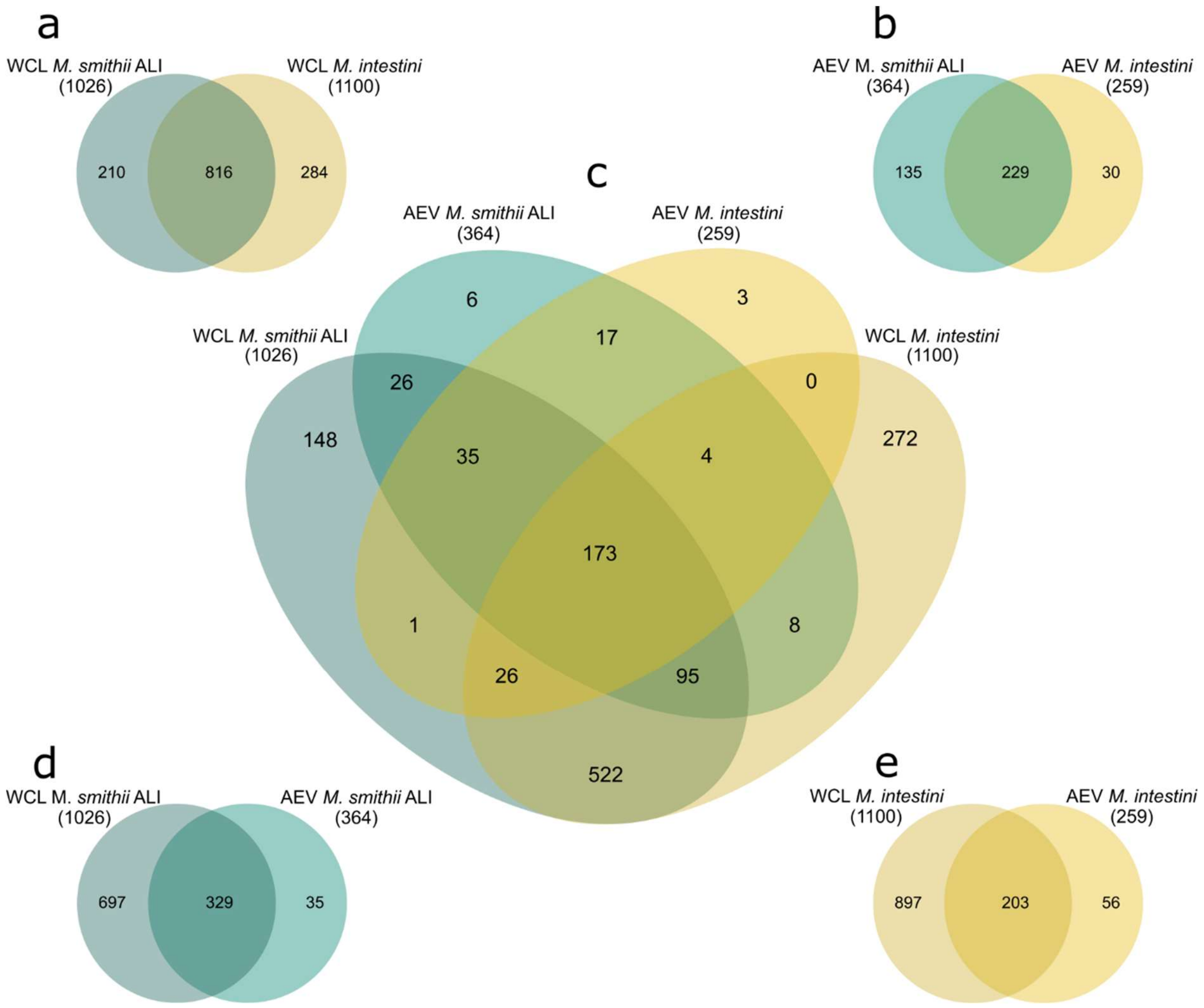

Supplementary Figure S2: Venn diagrams constructed with proteins based on a prevalence in 3 of 3 replicates per group of WCL *M. smithii* ALI, WCL *M. intestini*, AEV *M. smithii* ALI or AEV *M. intestini*. (a) WCL *M. smithii* ALI vs. WCL *M. intestini*, (b) AEV *M. smithii* ALI vs. AEV *M. intestini*, (c) WCL *M. smithii* ALI vs. AEV *M. smithii* ALI vs. AEV *M. intestini* vs. WCL *M. intestini*, (d) WCL *M. smithii* ALI vs. AEV *M. smithii* ALI, and (e) WCL *M. intestini* vs. AEV *M. intestini*. Abbreviations: (WCL) Whole cell lysate; (AEV) archaeal extracellular vesicles.

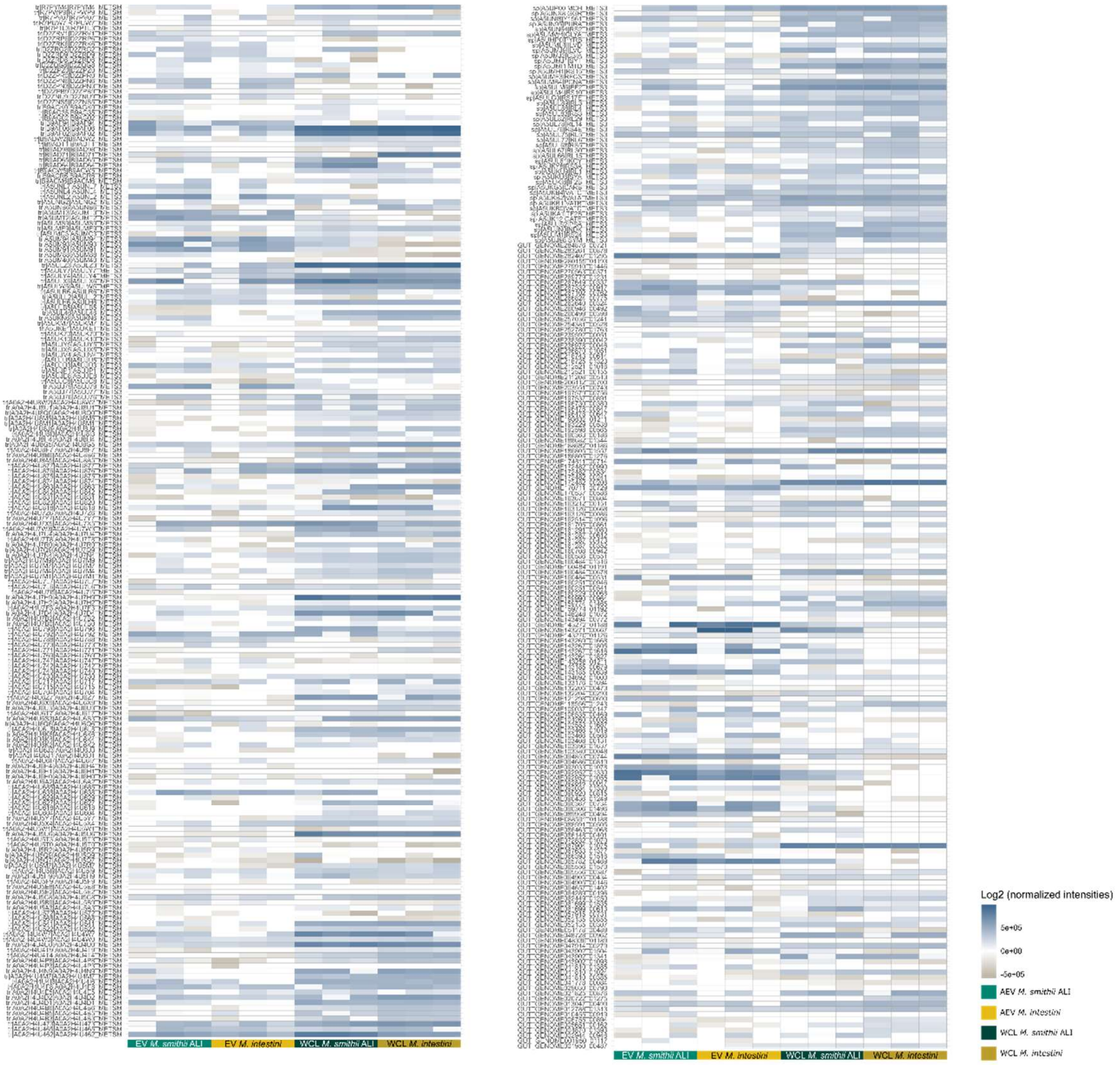

Supplementary Figure S3: The heatmap summarizes 229 proteins consistently identified across all six EV samples (3 replicates from AEV M. smithii ALI and 3 replicates from AEV *M. intestini*), with their annotations. Abbreviations: (WCL) Whole cell lysate; (AEV) archaeal extracellular vesicles.

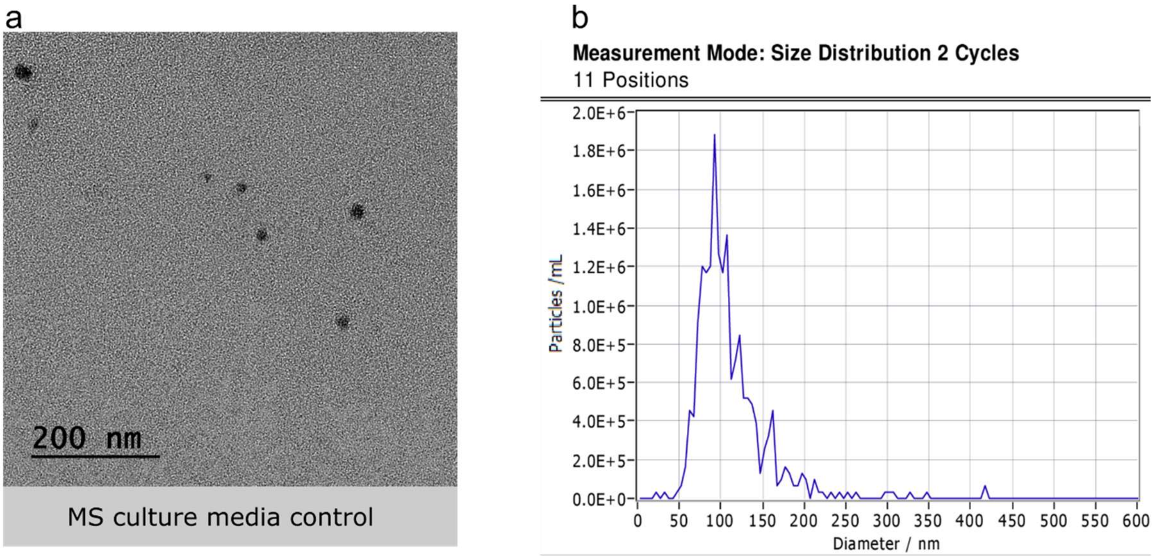

Supplementary Figure S4: (a) Representative negative staining electron microscopy image of MS culture media control, showing no present vesicles. (b) Representative NTA graph for the determination of AEV concentration. Measured strain: M. smithii ALI.

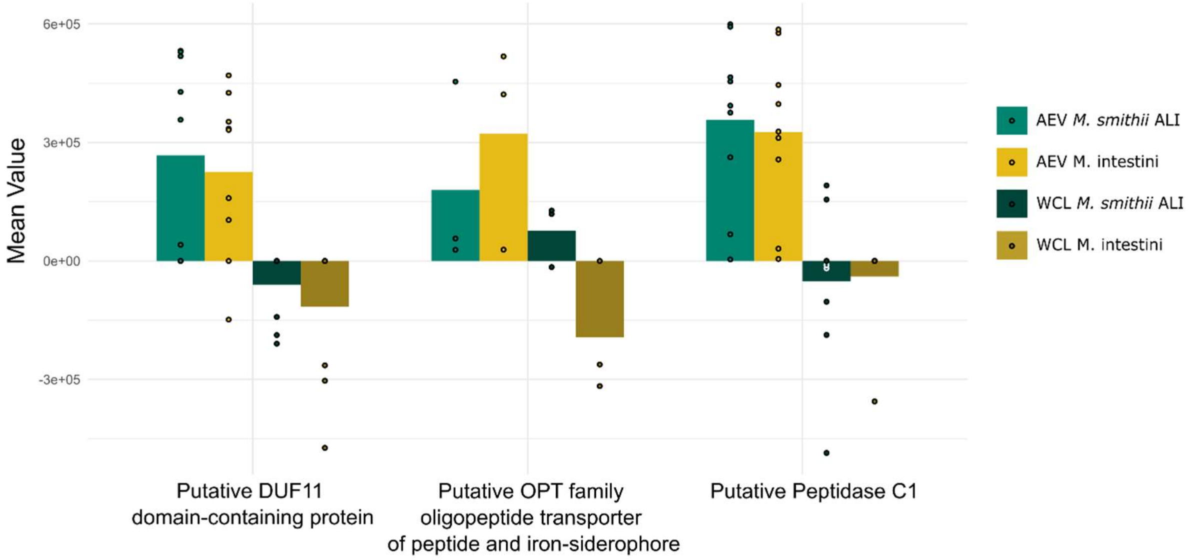

Supplementary Figure S5: Bar chart showing the mean intensities/relative abundances of selected protein groups in EVs and WCLs. Only proteins detected in all three biological replicates of both *M. smithii* ALI and M. intestini (n=229) are included in the analysis (Data: Supplementary Table 4). Individual data points reflect single proteins (mean across three biological replicates for each type of AEVs and WCLs).

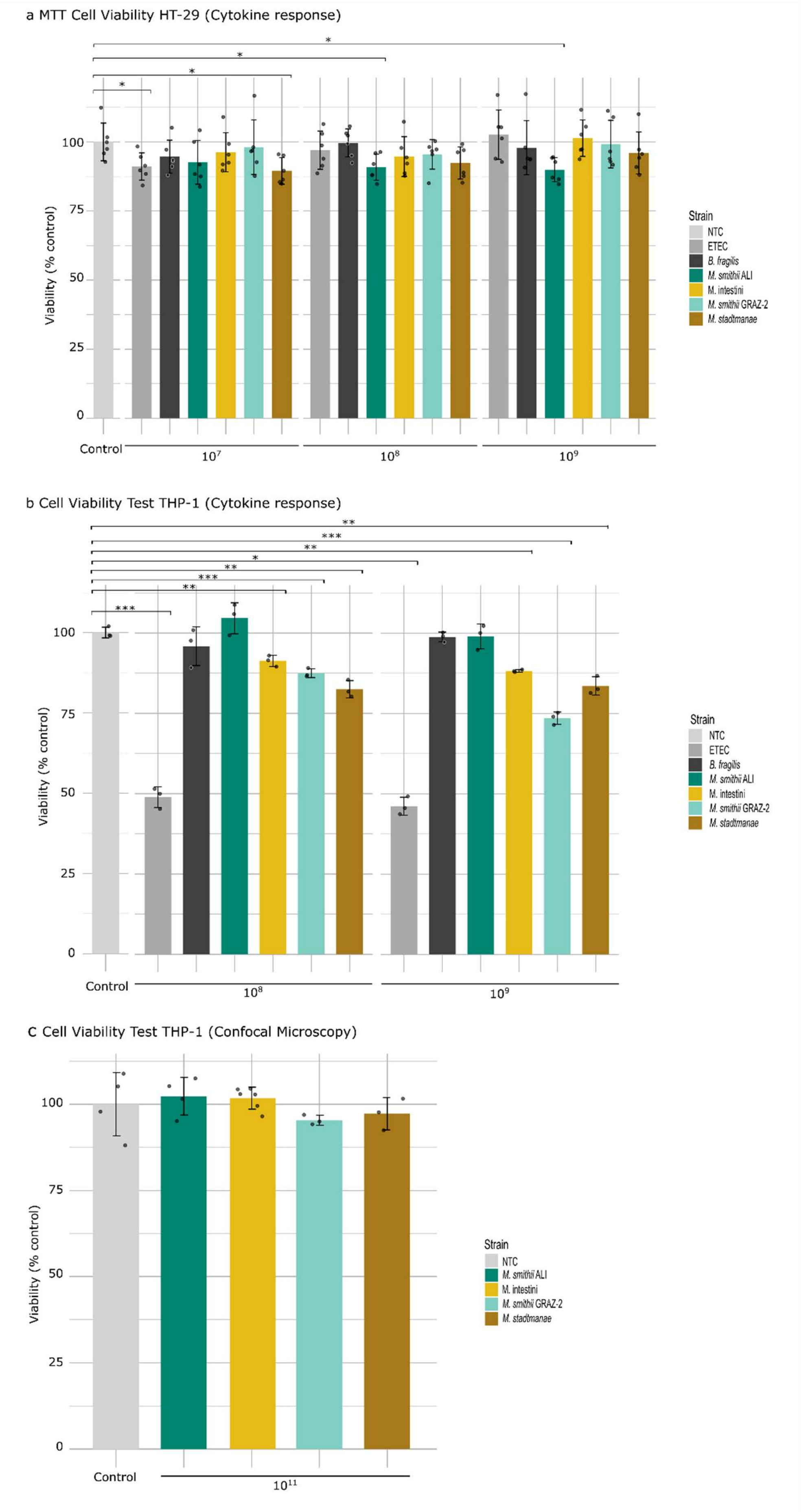
a MTT Cell Viability HT-29 (Cytokine response)
Viability (% control)
0
25
50
75
100
*
*
*
*
Control
10^7
10^8
10^9
Strain
NTC
ETEC
B. fragilis
M. smithii ALI
M. intestini
M. smithii GRAZ-2
M. stadtmanae
b Cell Viability Test THP-1 (Cytokine response)
Viability (% control)
0
25
50
75
100
**
***
**
*
**
***
**
***
Control
10^8
10^9
Strain
NTC
ETEC
B. fragilis
M. smithii ALI
M. intestini
M. smithii GRAZ-2
M. stadtmanae
c Cell Viability Test THP-1 (Confocal Microscopy)
Viability (% control)
0
25
50
75
100
Control
10^11
Strain
NTC
M. smithii ALI
M. intestini
M. smithii GRAZ-2
M. stadtmanae

Supplementary Figure S6: (a) Cell viability test (MTT) of HT-29 cells after the co-incubation with bacterial and archaeal EVs originated from ETEC, *B. fragilis*, *M. smithii* ALI, *M. intestini*, *M. smithii* GRAZ-2, and *M. stadtmanae*. Incubation time 20h for testing the cytokine response of HT-29 cells. Three doses of EVs were tested: 10^7, 10^8, and 10^9 particles/ml. T-test or Wilcoxon test was used based on the normal distribution of the data. (b) Cell viability test of THP-1 cells after they were incubated with bacterial and archaeal EVs originated from ETEC, *B. fragilis*, *M. smithii* ALI, *M. intestini*, *M. smithii* GRAZ-2, and *M. stadtmanae*. Time of incubation was 24h to test the cytokine response of THP-1 cells. Two doses of EVs were tested: 10^8, and 10^9 particles/ml. To determine significant differences t-test was used. (c) Cell viability test of THP-1 test during the preparation of confocal immunofluorescence microscopy. Cells were incubated with archaeal EVs from *M. smithii* ALI, *M. intestini*, *M. smithii* GRAZ-2, and *M. stadtmanae*. One dose of EVs was tested: 10^{11} particles/ml. To determine significant differences t-test was used. (Supplementary Table 13).

Individual data points reflect each replicate. Number on the bottom of each figure indicates the dose of EVs exposed to the cell lines. Significant differences are highlighted with an asterisk. Abbreviations: No treatment control (NTC); Enterotoxigenic Escherichia coli (ETEC). One asterisk represents $p < 0.05$, ** represents $p < 0.01$, and *** represents $p < 0.001$.
