# Supplementary material for: Proteomic and metabolomic profiling of extracellular vesicles produced by human gut archaea
Source: Nat Commun. 2025 Jun 3;16:5094. doi: 10.1038/s41467-025-60271-w (PMC12134236; doi:10.1038/s41467-025-60271-w)
Supplement: Supplementary file 2 — Description of Additional Supplementary Files [file 41467_2025_60271_MOESM2_ESM.pdf]

## Description of Additional Supplementary Files:

**Supplementary Data 1:** Summary of archaeal vesicle properties, including size, concentration, protein, DNA, RNA, and lipid content. Protein, DNA, RNA, and lipid contents were normalized to μg/$10^{10}$ particles. Data are presented as mean, minimum (Min.), and maximum (Max.) values for properties with multiple replicates. Single values are shown where only one measurement is available (e.g., lipid content). Biological replicates: M. smithii ALI = 9; M. intestini = 12; M. smithii GRAZ-2 = 5; M. stadtmanae = 6. Statistical analysis details are provided in the main text: One-way ANOVA was performed for all parameters except DNA and lipid content, with post hoc Tukey's HSD tests where applicable. Significant differences were observed for size between M. smithii ALI and M. intestini ($P = 0.046$), as well as M. intestini and M. smithii GRAZ-2 ($P = 0.007$). No significant differences were found for concentration, protein, DNA, RNA, and lipid content.

**Supplementary Data 2:** Raw data of vesicle properties (Concentration, size, DNA, RNA, Protein, and lipid content), including mean, min and max values.

**Supplementary Data 3:** Vesicle properties: Normalization of protein, DNA, and RNA vesicle content [μg/10^10 particles].

**Supplementary Data 4:** Proteomics: Raw data protein intensities/relative abundance.

**Supplementary Data 5:** Summary of identified proteins in proteomics analysis.

**Supplementary Data 6:** Proteomics: Intensities/relative abundances of all proteins found in all biological replicates of vesicles of M. smithii ALI (Ali) and M. intestini (int), in vesicle preparations (EV) and whole cell lysates (WCL).

**Supplementary Data 7:** Proteomics: (Putative) ALP's, their representative sequences, transmembrane helices, and annotations through InterPro.

**Supplementary Data 8:** Proteomics: Proteins found in all biological replicates (n=3+3) and both species (Methanobrevibacter intestini and M. smithii ALI) were annotated through the archaeome protein catalogue (Chibani et al., 2022) and pfam classification. Categorization followed mostly UNIPROT categories (biological processes) whenever possible. For the sake of visualization, additional grouping was performed as indicated in the last column.

**Supplementary Data 9:** Annotation of proteomics experiment for comparing membrane fractions of M. smithii ALI and M. intestini to AEVs. With a focus on Adhesins/Adhesin-like proteins (ALPs). 'Membrane fraction only' is the combination of ALPs found in M. smithii ALI and M. intestini; 'M. smithii ALI only' and 'M. intestini only' are summaries of ALPs for each strain.

**Supplementary Data 10:** Metabolomics: Metabolites found in internal database for mass spectrometry derived AEV metabolomics. log2 fold changes, as well as p-values, were calculated by the Compound Discoverer software (Tukey HSD test (posthoc), after an analysis of variance (ANOVA) test), which applies a correction for multiple comparisons. All tests were two-sided.

**Supplementary Data 11:** Raw data Luminex Immunoassay of THP-1 and HT-29 cells upon exposure to AEVs (M. smithii ALI, M. intestini, M. smithii GRAZ-2, and M. stadtmanae) and BEVs (ETEC, B. fragilis).

**Supplementary Data 12:** Luminex Immunoassay Mean calculation and log10 transformation of THP-1 and HT-29 cells upon exposure to AEVs (M. smithii ALI, M. intestini, M. smithii GRAZ-2, and M. stadtmanae) and BEVs (ETEC, B. fragilis).

**Supplementary Data 13:** Cytotoxicity tests of HT-29 and differentiated THP-1 cells upon exposure to AEVs (M. smithii ALI, M. intestini, M. smithii GRAZ-2, and M. stadtmanae) and BEVs (ETEC, B. fragilis).
