# Supplementary material for: Proteomic and metabolomic profiling of extracellular vesicles produced by human gut archaea
Source: Nat Commun. 2025 Jun 3;16:5094. doi: 10.1038/s41467-025-60271-w (PMC12134236; doi:10.1038/s41467-025-60271-w)
Supplement: Supplementary file 6 — Source Data [file 41467_2025_60271_MOESM6_ESM.zip › SOURCE_DATA/Supplementary_figure_S4/representative NTA_graph_M.smithii_ALI.pdf]

Electrophoresis & Brownian Motion
Video Analysis
Laser Scattering Microscopy

www.particle-metrix.de

Operator (Report): ZetaView

Video Operator: ZetaView

Sample Parameters

Sample Name: ali2_29112023
Comment: Sample Remarks0: IZON qEV1 F1-4 pooled
Sample Remarks1:
Sample Remarks2:
Electrolyte: H_2O
Temperature: 24.55 °C sensed
pH 7.0 entered
Conductivity: 26.88 µS/cm sensed

Instrument Parameters

Laser Wavelength: 488 nm
Filter Wavelength: Scatter

Measurement Parameters

Cell S/N: ZNTA

Measurement Mode: Size Distribution 2 Cycles

11 Positions

Result (sizes in nm)

	Number	Concentration	Volume
Median (X50)	98.3	98.3	1768.7
Span	94.5	94.4	557.0

Concentration: 1.8E+7 Particles / mL
Dilution Factor: 1000
Original Concentration: 1.8E+10 Particles / mL

Quality

Average Counted Particles per Frame: 50
Number of Traced Particles: 555

Analysis Parameters

Max Area: 2000, Min Area: 5, Min Brightness: 20

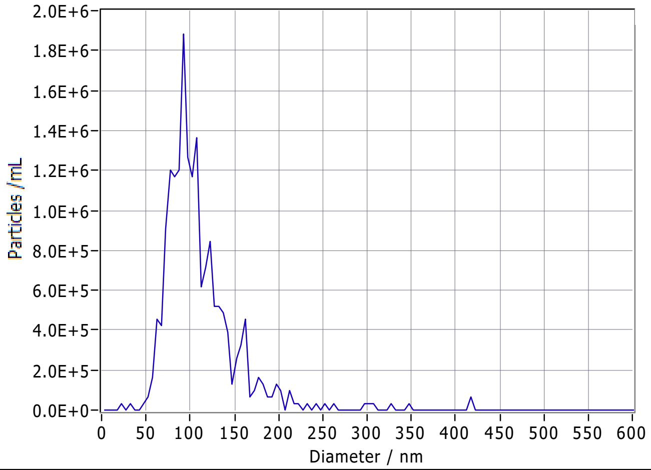

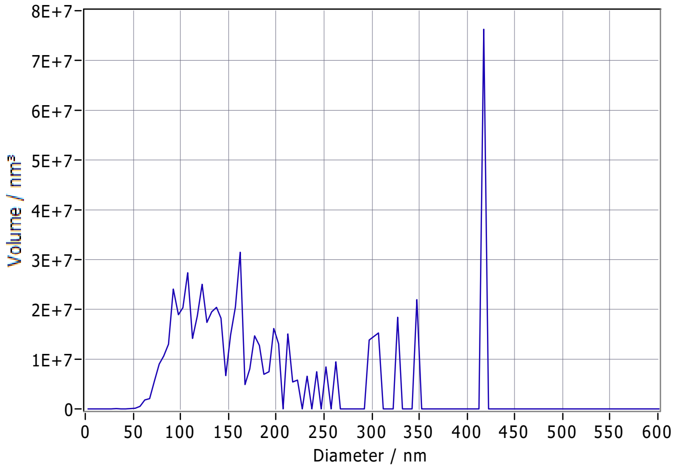

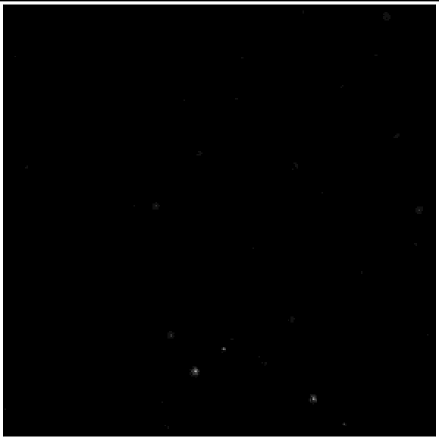

Peak Analysis (Concentration)

Diameter / nm	Particles/mL	FWHM / nm	Percentage
93.2	1.5E+6	46.1	100.0

X Values (all sizes are given in nm)

	Number	Concentration	Volume
X10	70.8	70.8	215.5
X50	98.3	98.3	1768.7
X90	158.0	158.0	1771.7
Span	0.9	0.9	0.9
Mean	115.8	115.8	1428.9
StdDev	94.5	94.4	557.0

Comment

(Signature)

Analyzed Video: Z:\CARDING\Viktoria\20231129_0006_ali2_29112023_size_488.avi

ZetaVIEW S/N 20-534, Software ZetaView (version 8.05.12 SP1), Camera 0.713 mum/px

Experiment: 2023-11-29 09:55, Report: 2023-11-29 09:58
