# Supplementary figures and images for: Proteomic and metabolomic profiling of extracellular vesicles produced by human gut archaea
Source: Nat Commun. 2025 Jun 3;16:5094. doi: 10.1038/s41467-025-60271-w (PMC12134236; doi:10.1038/s41467-025-60271-w)

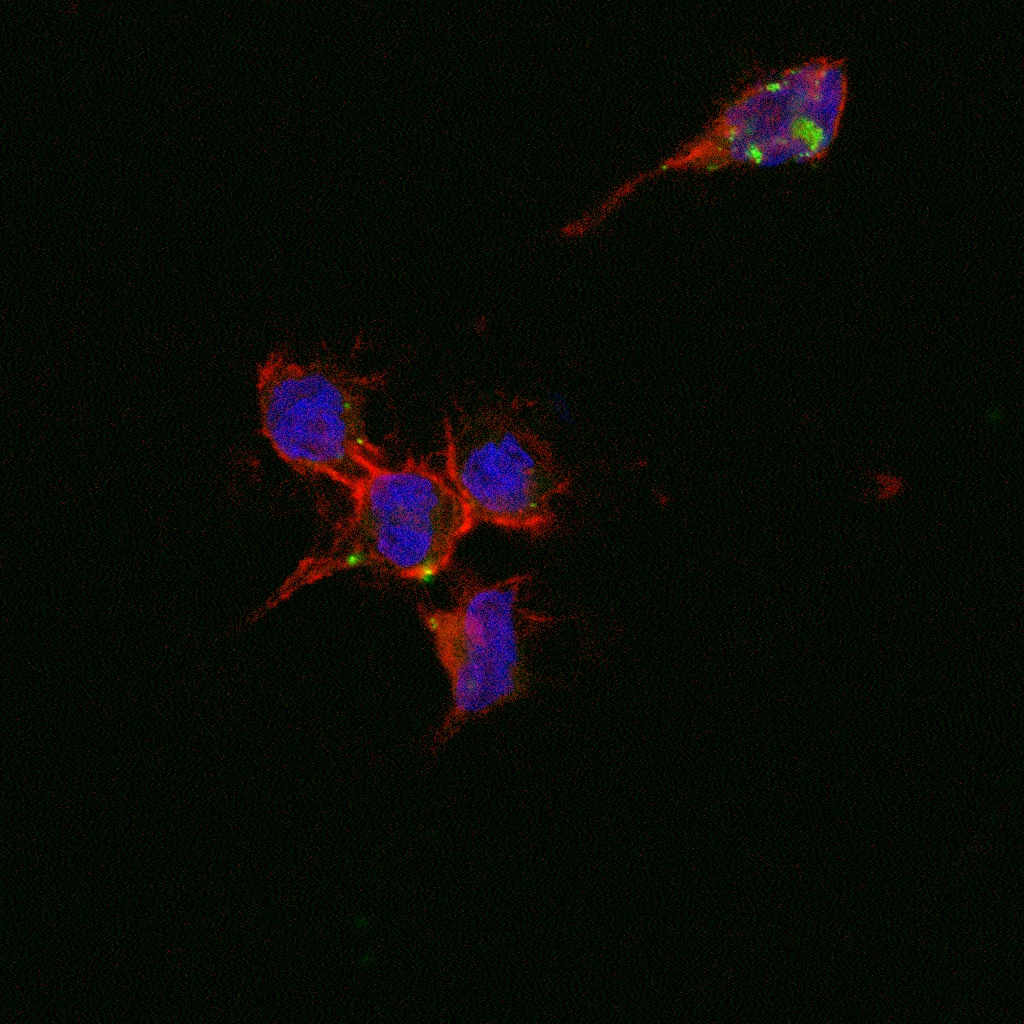

Supplement: Supplementary file 6 — Source Data [file 41467_2025_60271_MOESM6_ESM.zip › SOURCE_DATA/Confocal_microscopy_images_originals_figure_5/M. intestini_Fig_5b/edited_Image 64_int_c1-3.jpg]

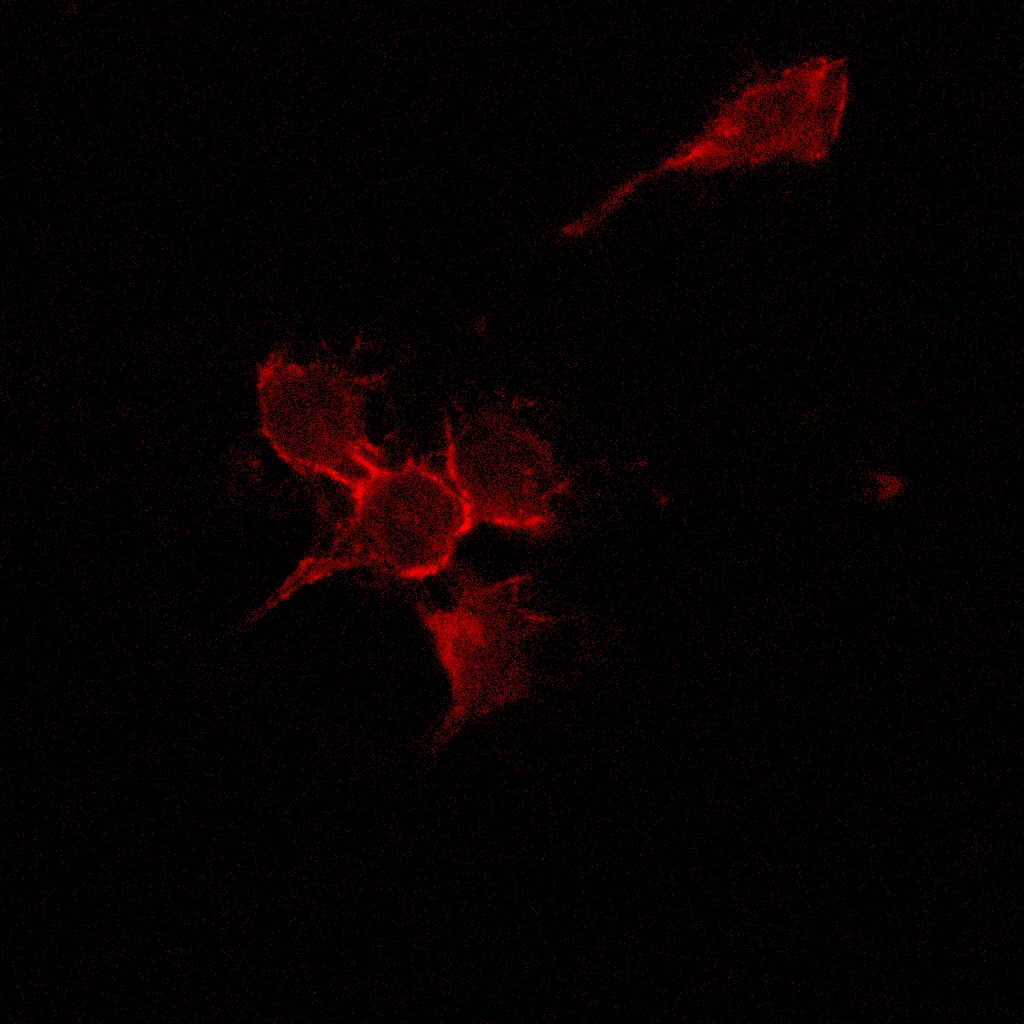

Supplement: Supplementary file 6 — Source Data [file 41467_2025_60271_MOESM6_ESM.zip › SOURCE_DATA/Confocal_microscopy_images_originals_figure_5/M. intestini_Fig_5b/edited_Image 64_int_c1.jpg]

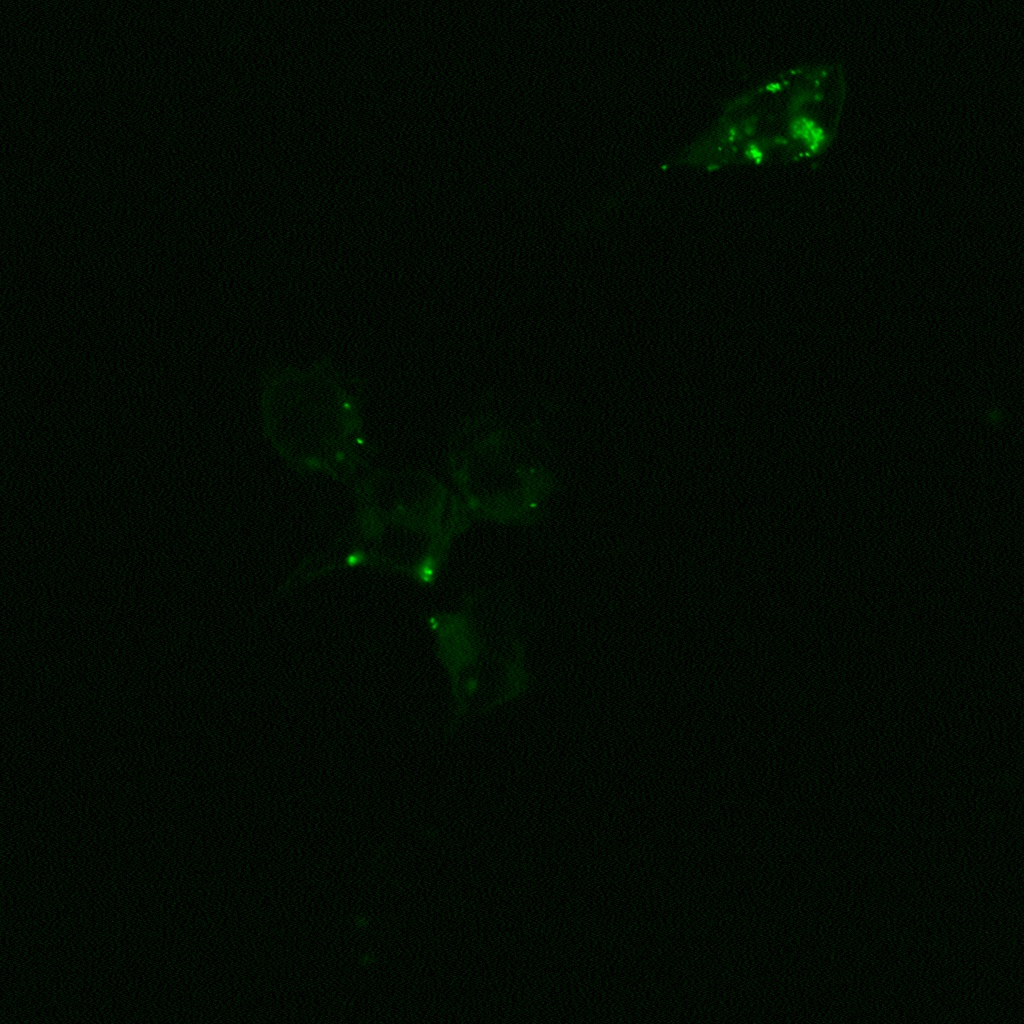

Supplement: Supplementary file 6 — Source Data [file 41467_2025_60271_MOESM6_ESM.zip › SOURCE_DATA/Confocal_microscopy_images_originals_figure_5/M. intestini_Fig_5b/edited_Image 64_int_c2.jpg]

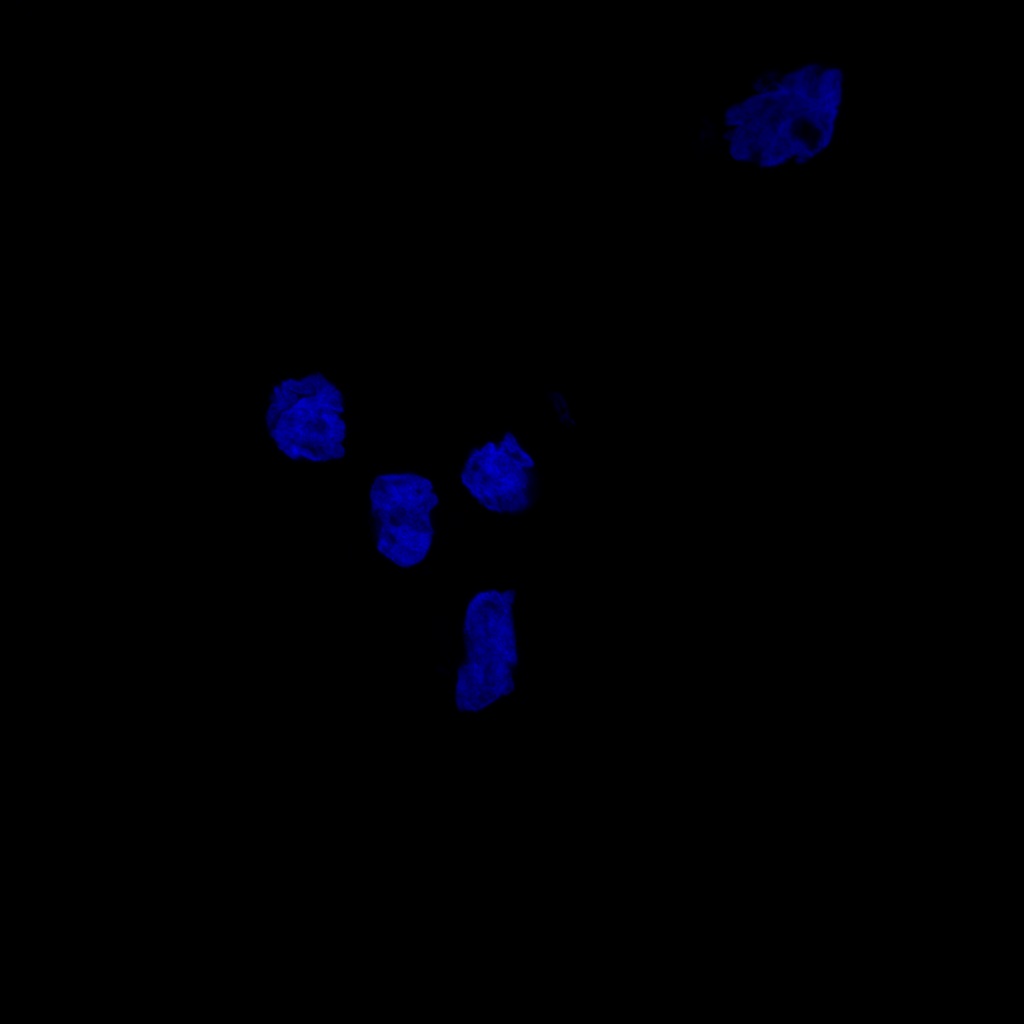

Supplement: Supplementary file 6 — Source Data [file 41467_2025_60271_MOESM6_ESM.zip › SOURCE_DATA/Confocal_microscopy_images_originals_figure_5/M. intestini_Fig_5b/edited_Image 64_int_c3.jpg]

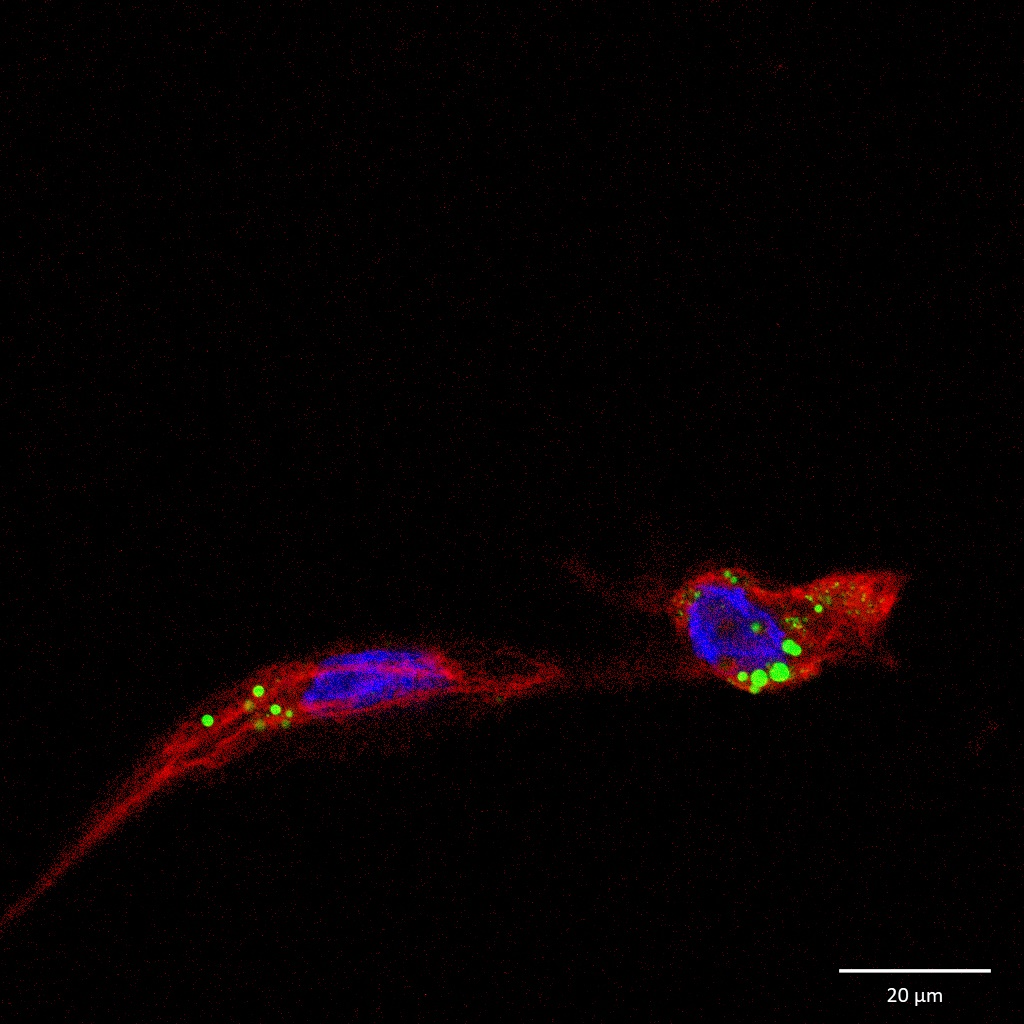

Supplement: Supplementary file 6 — Source Data [file 41467_2025_60271_MOESM6_ESM.zip › SOURCE_DATA/Confocal_microscopy_images_originals_figure_5/M. smithii ALI_Fig_5a/edited_Image 38_Ali_2.0_c1-3.jpg]

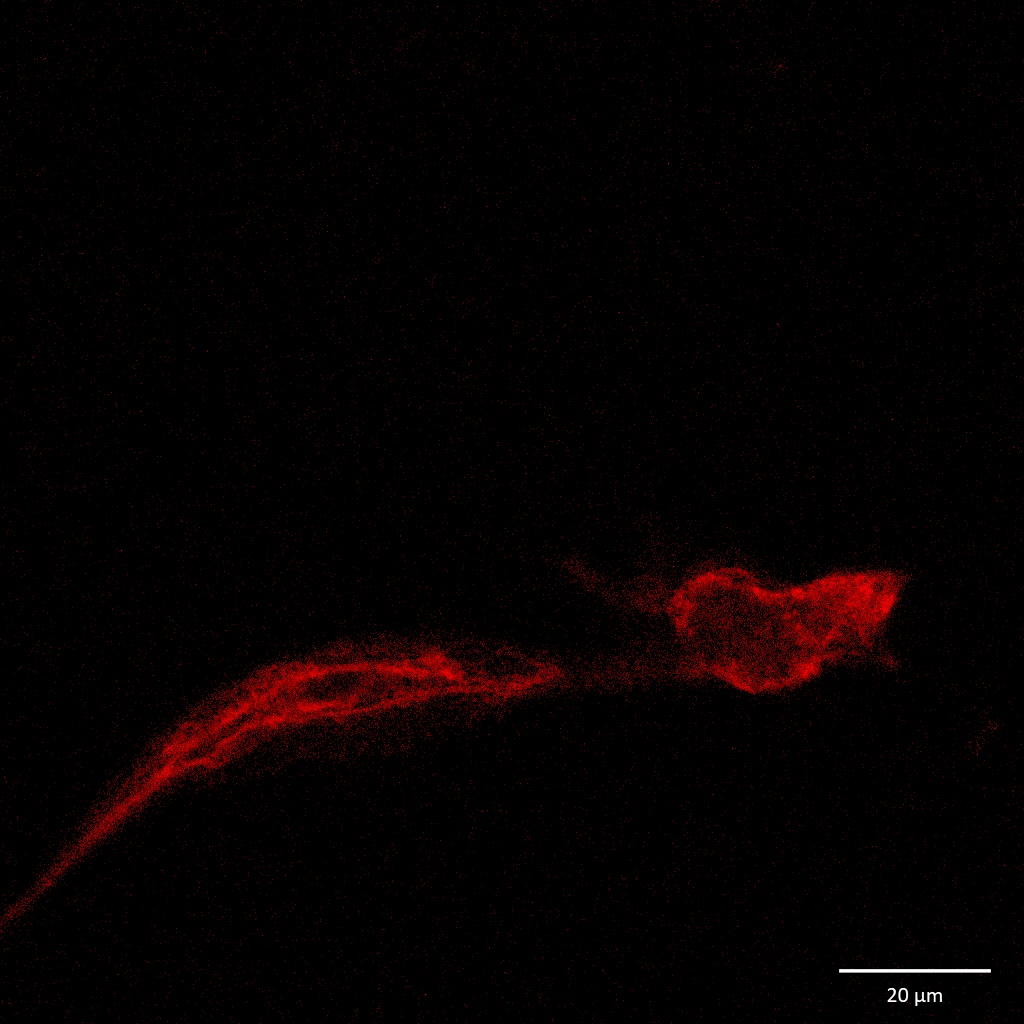

Supplement: Supplementary file 6 — Source Data [file 41467_2025_60271_MOESM6_ESM.zip › SOURCE_DATA/Confocal_microscopy_images_originals_figure_5/M. smithii ALI_Fig_5a/edited_Image 38_Ali_2.0_c1.jpg]

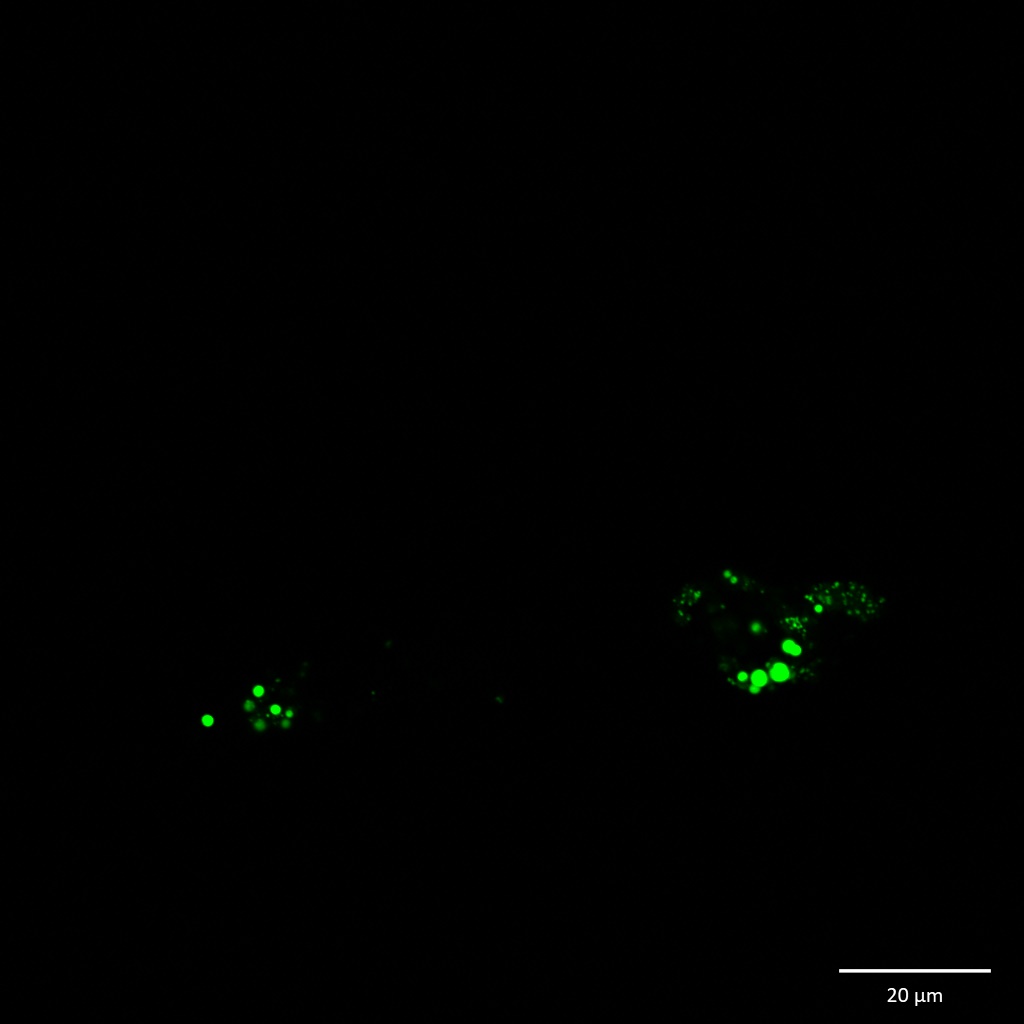

Supplement: Supplementary file 6 — Source Data [file 41467_2025_60271_MOESM6_ESM.zip › SOURCE_DATA/Confocal_microscopy_images_originals_figure_5/M. smithii ALI_Fig_5a/edited_Image 38_Ali_2.0_c2.jpg]

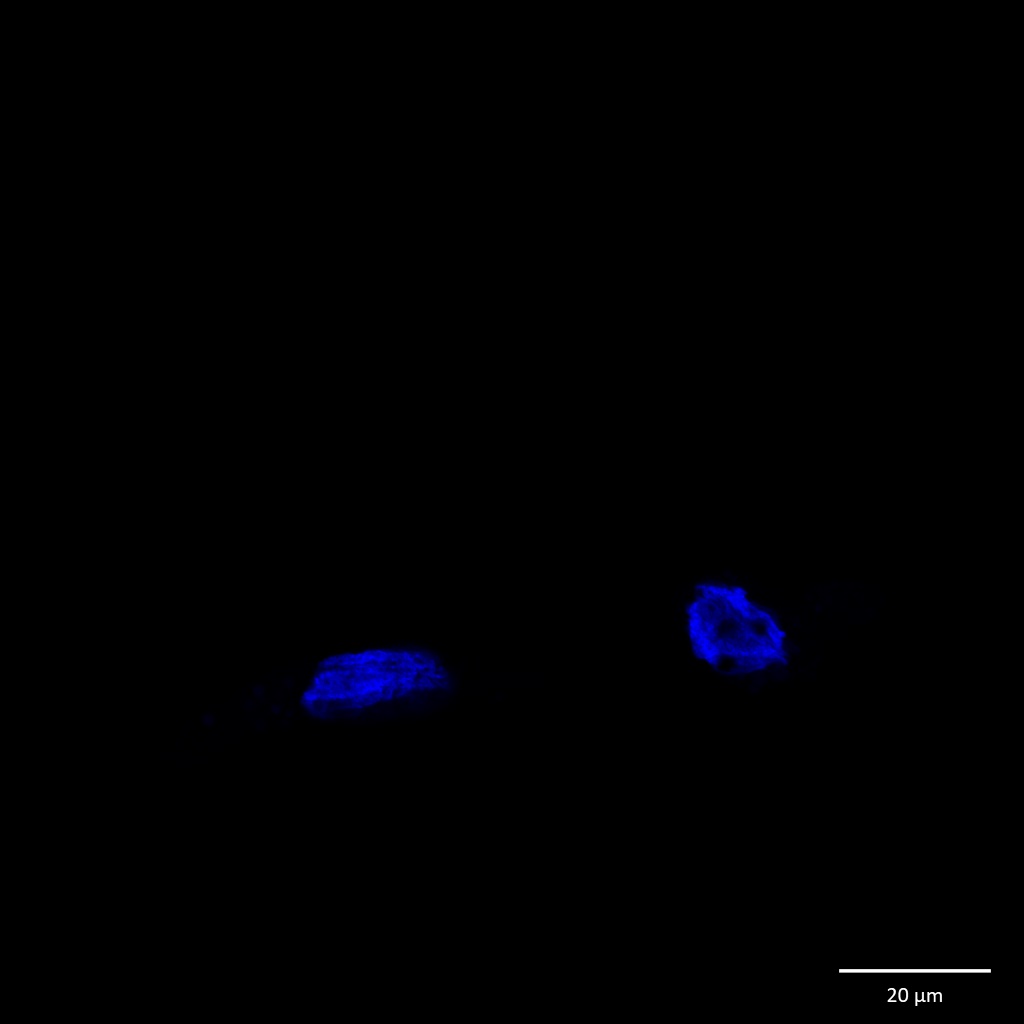

Supplement: Supplementary file 6 — Source Data [file 41467_2025_60271_MOESM6_ESM.zip › SOURCE_DATA/Confocal_microscopy_images_originals_figure_5/M. smithii ALI_Fig_5a/edited_Image 38_Ali_2.0_c3.jpg]

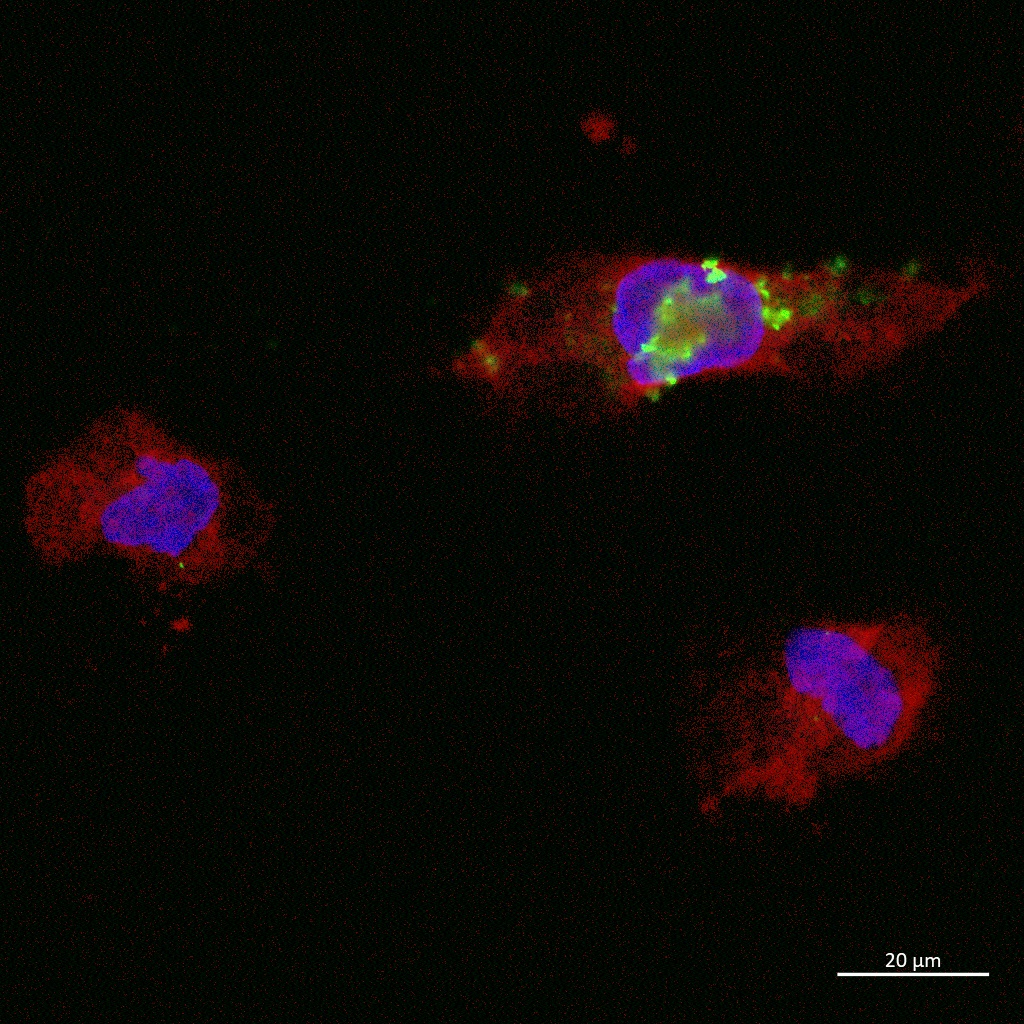

Supplement: Supplementary file 6 — Source Data [file 41467_2025_60271_MOESM6_ESM.zip › SOURCE_DATA/Confocal_microscopy_images_originals_figure_5/M. smithii GRAZ-2_Fig.5c/editedImage 28_GRAZ-2_2.0_c1-3.jpg]

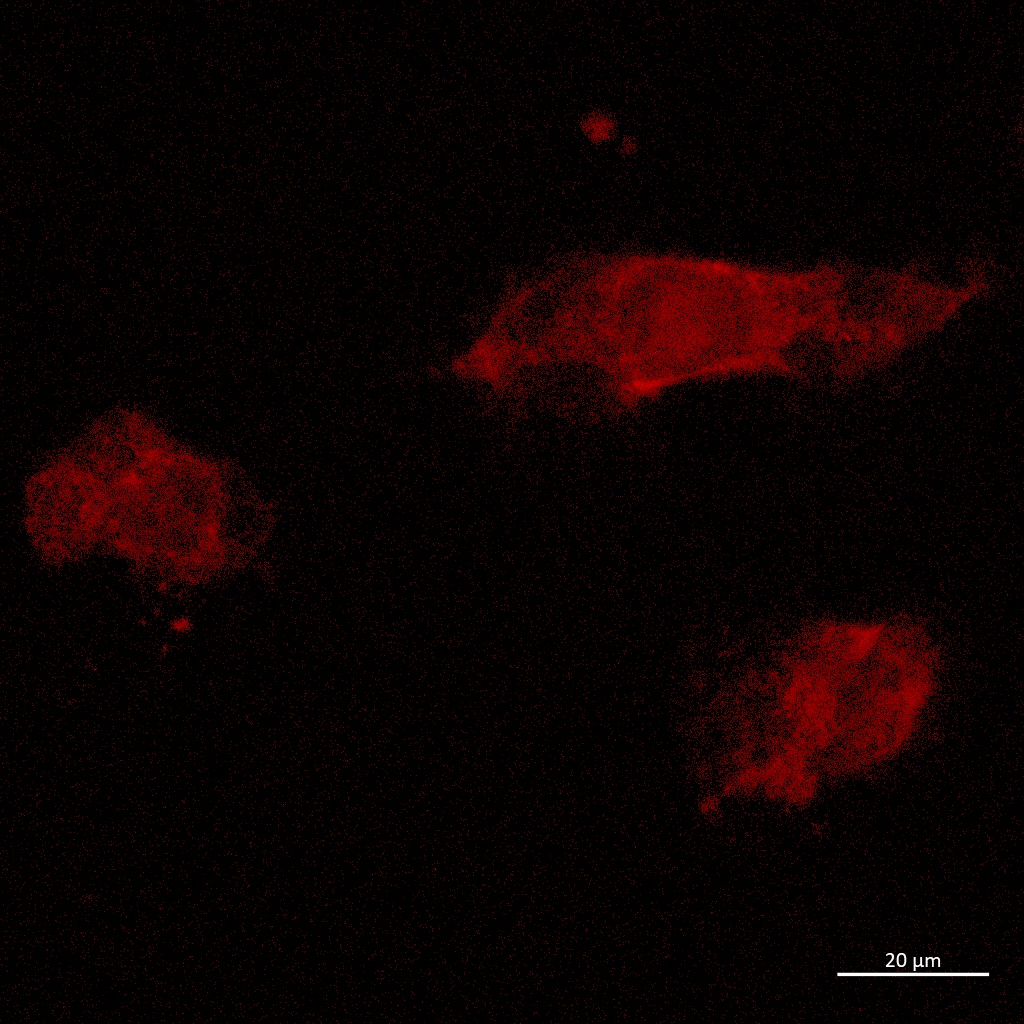

Supplement: Supplementary file 6 — Source Data [file 41467_2025_60271_MOESM6_ESM.zip › SOURCE_DATA/Confocal_microscopy_images_originals_figure_5/M. smithii GRAZ-2_Fig.5c/editedImage 28_GRAZ-2_2.0_c1.jpg]

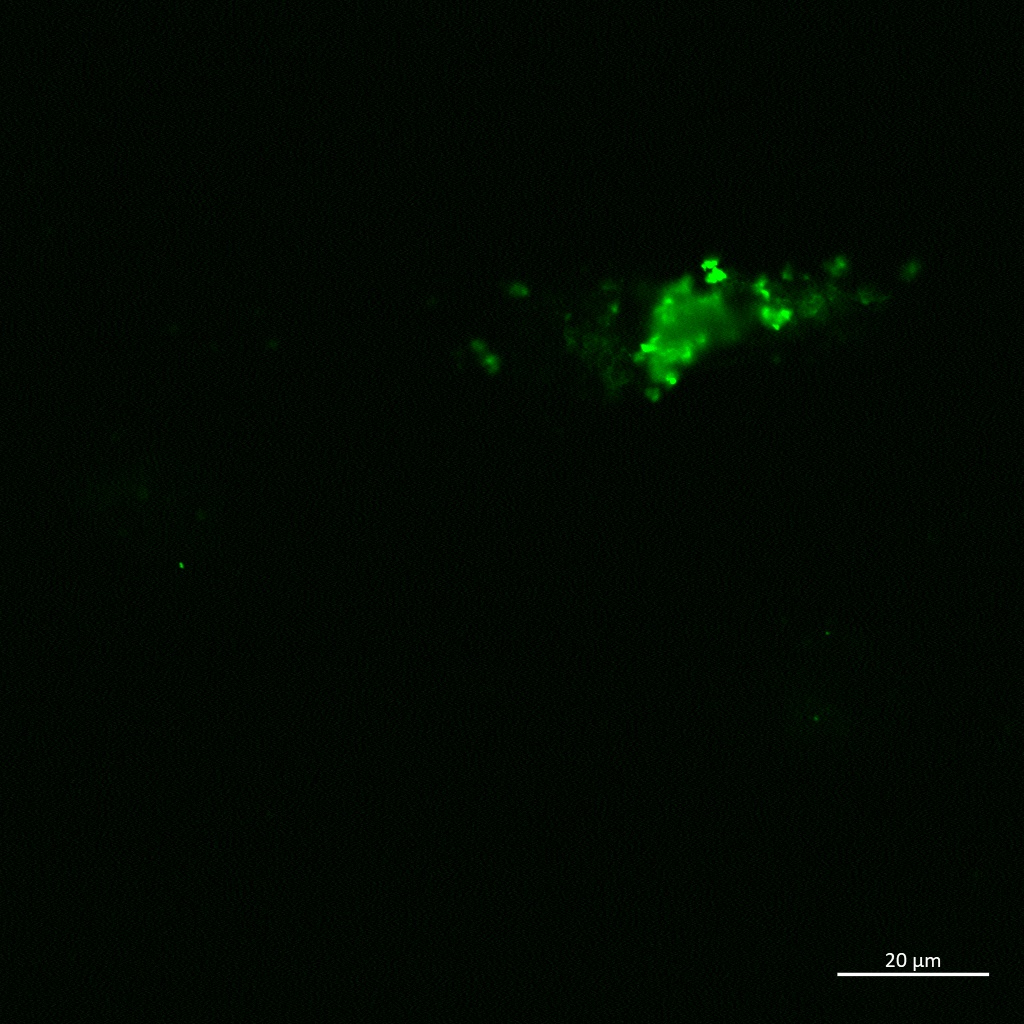

Supplement: Supplementary file 6 — Source Data [file 41467_2025_60271_MOESM6_ESM.zip › SOURCE_DATA/Confocal_microscopy_images_originals_figure_5/M. smithii GRAZ-2_Fig.5c/editedImage 28_GRAZ-2_2.0_c2.jpg]

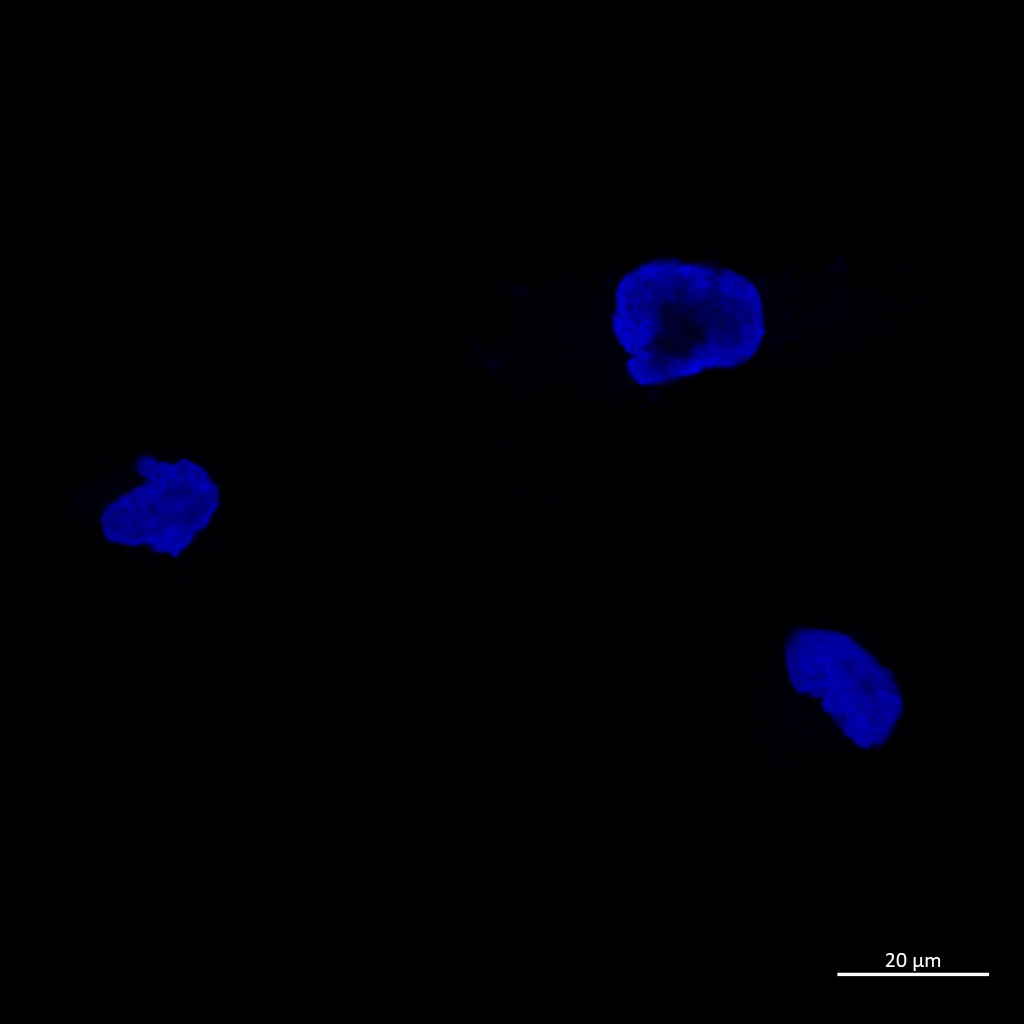

Supplement: Supplementary file 6 — Source Data [file 41467_2025_60271_MOESM6_ESM.zip › SOURCE_DATA/Confocal_microscopy_images_originals_figure_5/M. smithii GRAZ-2_Fig.5c/editedImage 28_GRAZ-2_2.0_c3.jpg]

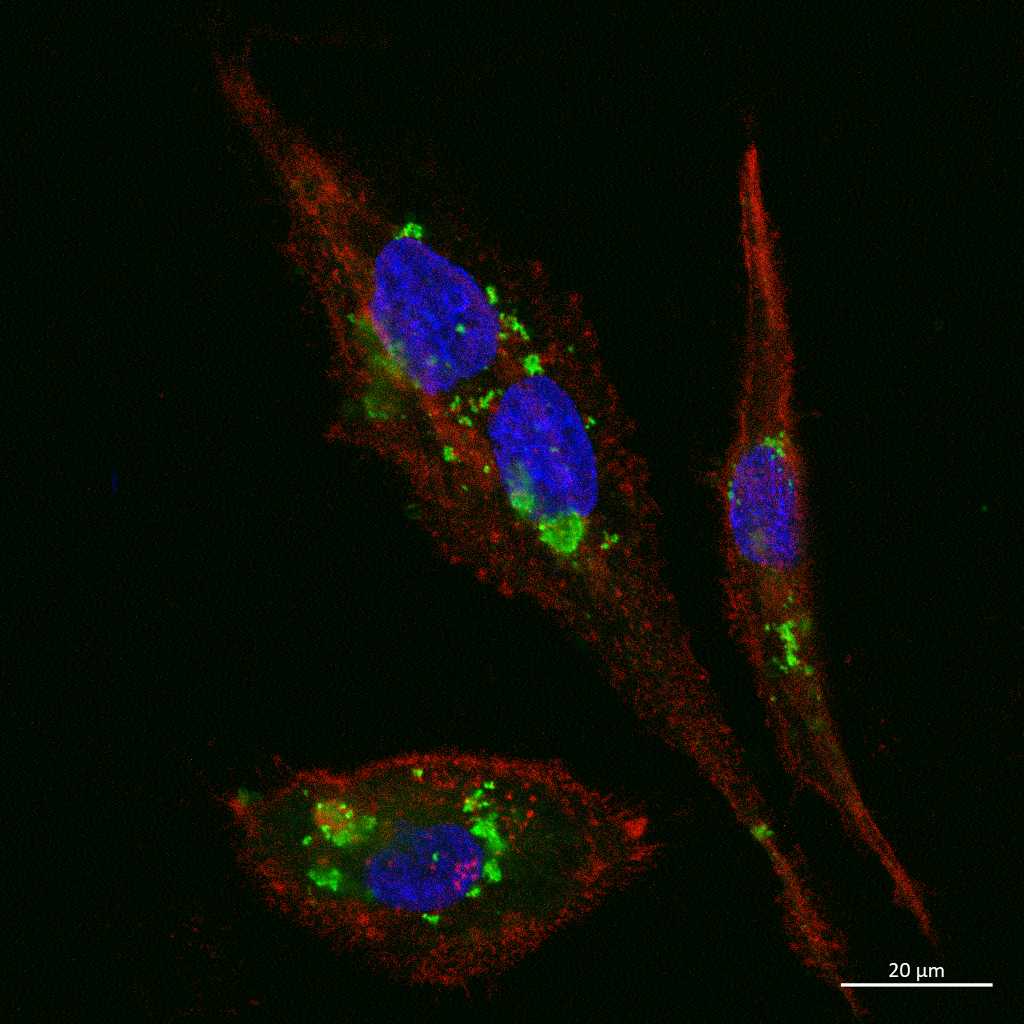

Supplement: Supplementary file 6 — Source Data [file 41467_2025_60271_MOESM6_ESM.zip › SOURCE_DATA/Confocal_microscopy_images_originals_figure_5/M. stadtmanae_Fig.5d/edited_stadtmanae_c1-3.tif]

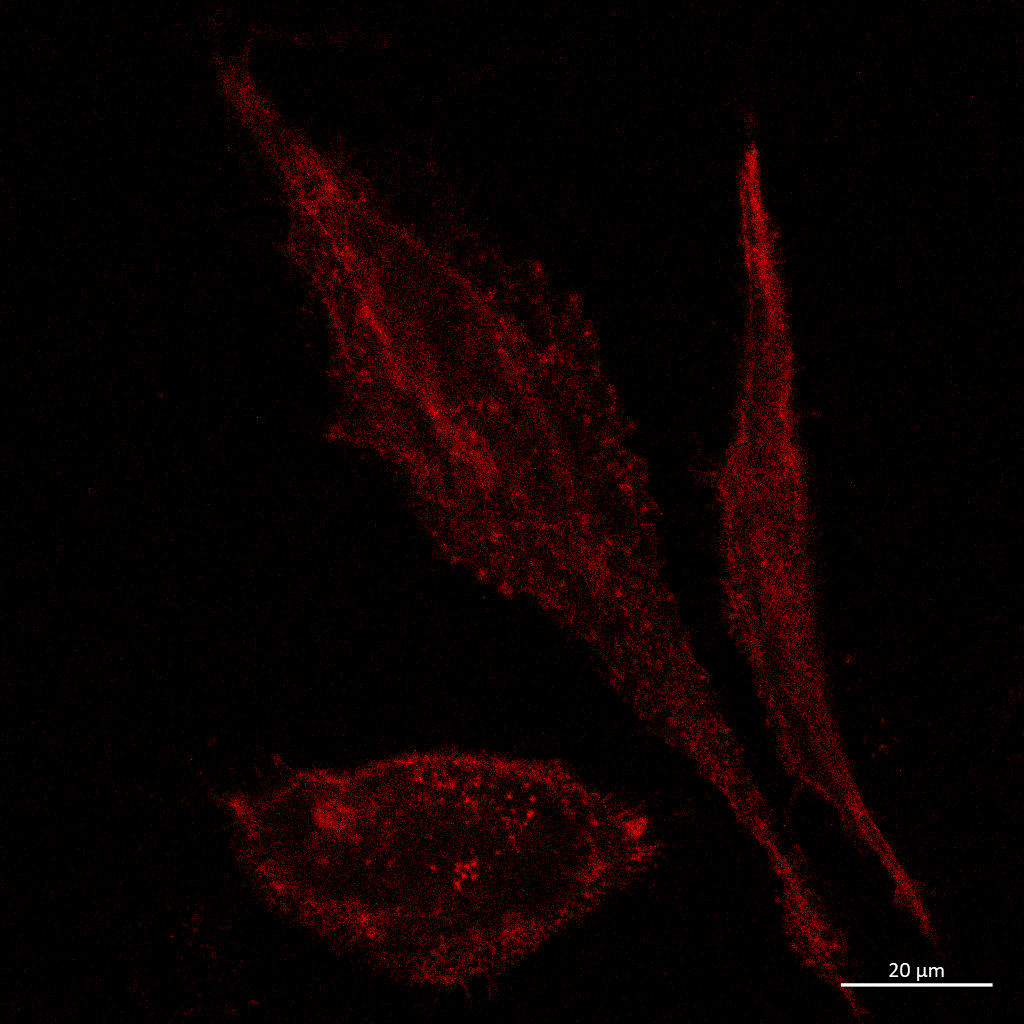

Supplement: Supplementary file 6 — Source Data [file 41467_2025_60271_MOESM6_ESM.zip › SOURCE_DATA/Confocal_microscopy_images_originals_figure_5/M. stadtmanae_Fig.5d/edited_stadtmanae_c1.tif]

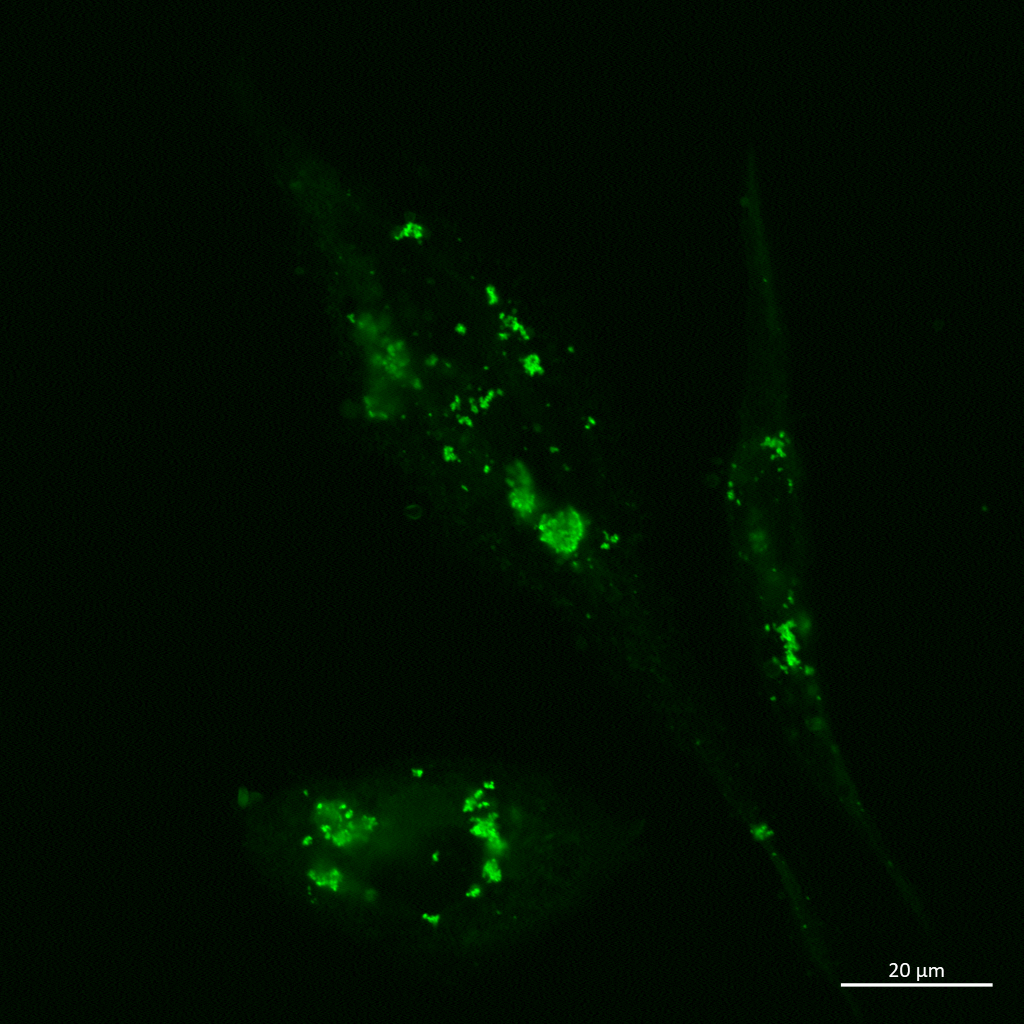

Supplement: Supplementary file 6 — Source Data [file 41467_2025_60271_MOESM6_ESM.zip › SOURCE_DATA/Confocal_microscopy_images_originals_figure_5/M. stadtmanae_Fig.5d/edited_stadtmanae_c2.tif]

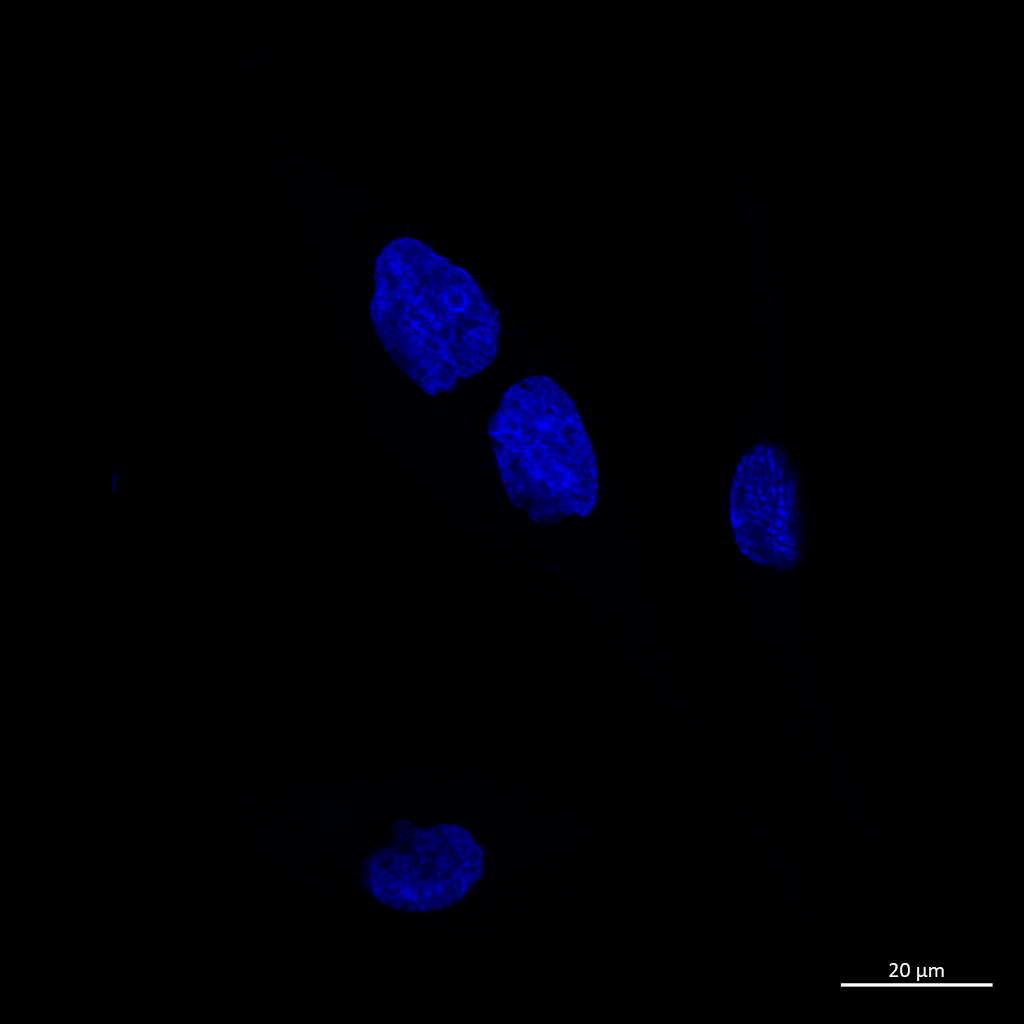

Supplement: Supplementary file 6 — Source Data [file 41467_2025_60271_MOESM6_ESM.zip › SOURCE_DATA/Confocal_microscopy_images_originals_figure_5/M. stadtmanae_Fig.5d/edited_stadtmanae_c3.tif]

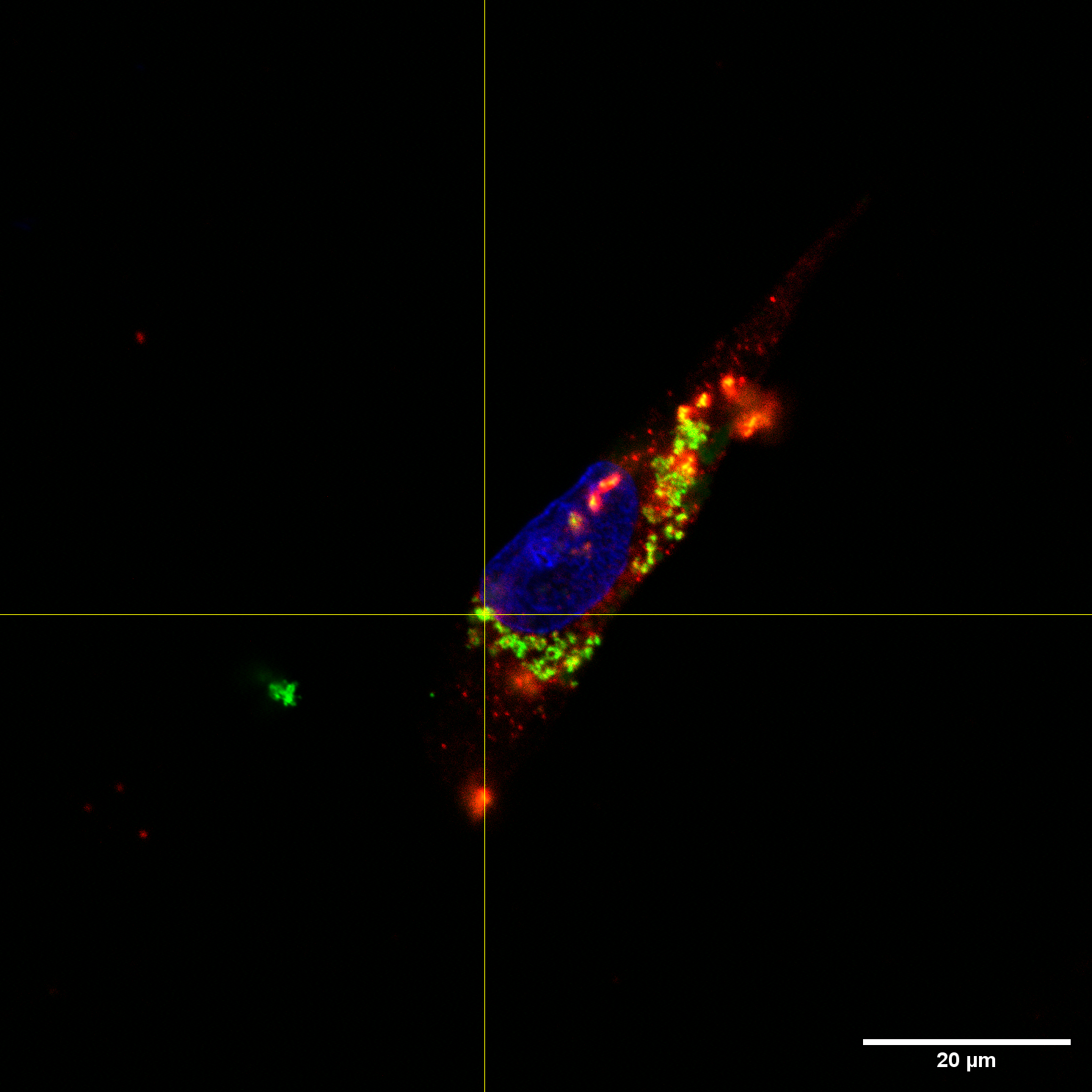

Supplement: Supplementary file 6 — Source Data [file 41467_2025_60271_MOESM6_ESM.zip › SOURCE_DATA/Confocal_microscopy_images_originals_figure_5/Z-stack_M.stadtmanae_Fig.5e/59-1.czi (RGB).tif v2.tif]

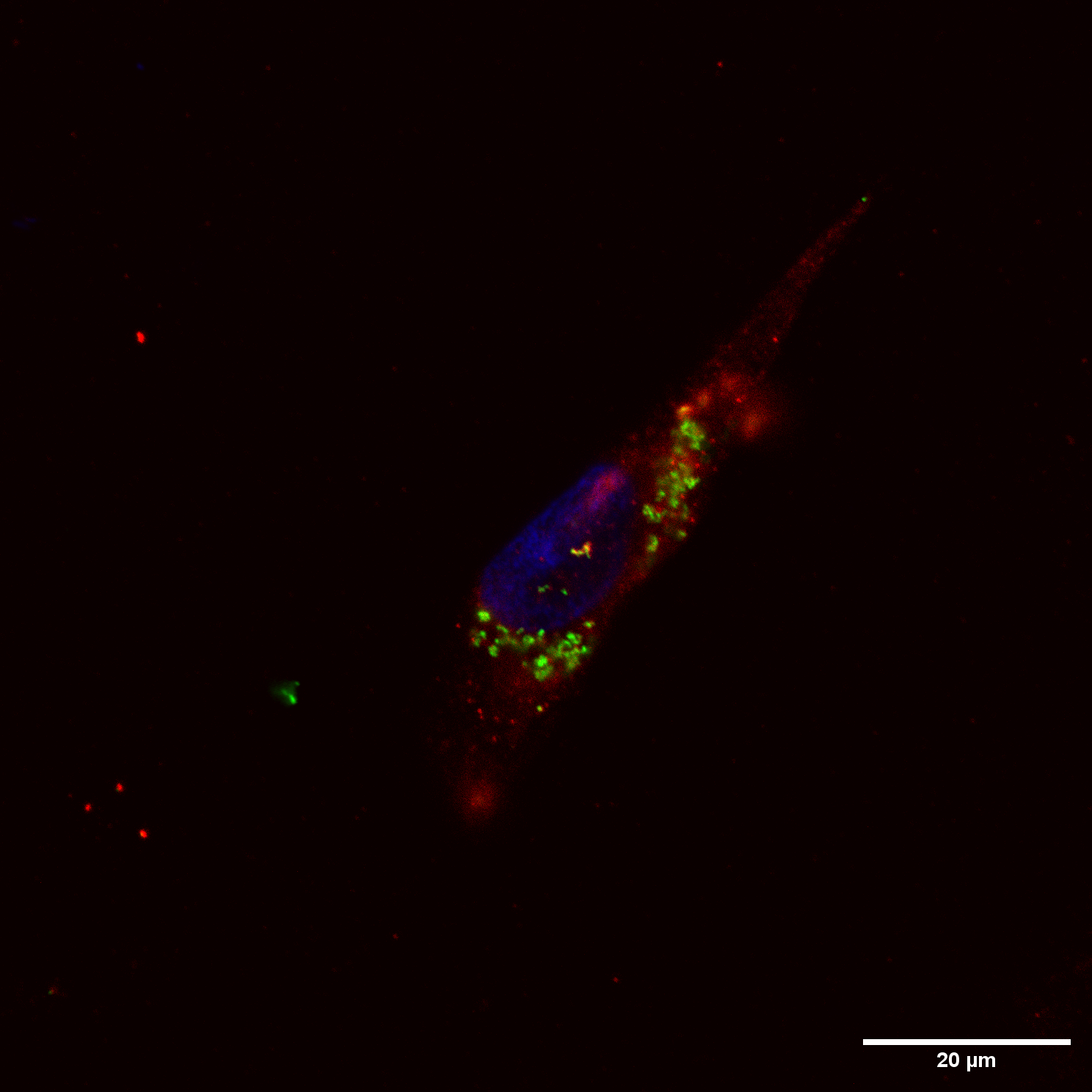

Supplement: Supplementary file 6 — Source Data [file 41467_2025_60271_MOESM6_ESM.zip › SOURCE_DATA/Confocal_microscopy_images_originals_figure_5/Z-stack_M.stadtmanae_Fig.5e/59-1.czi (RGB).tif z-slice 19.tif]

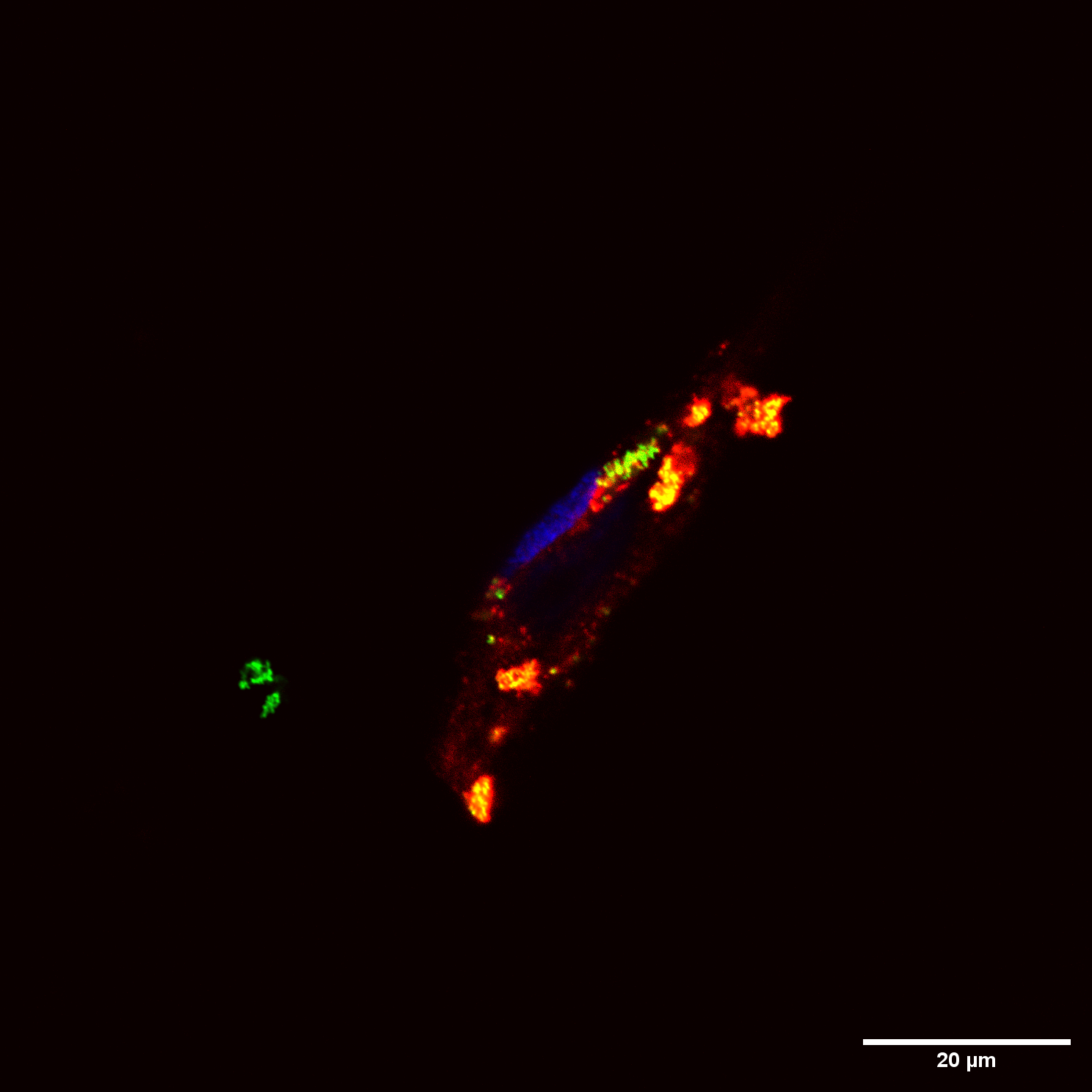

Supplement: Supplementary file 6 — Source Data [file 41467_2025_60271_MOESM6_ESM.zip › SOURCE_DATA/Confocal_microscopy_images_originals_figure_5/Z-stack_M.stadtmanae_Fig.5e/59-2.czi (RGB).tif z-slice 9.tif]

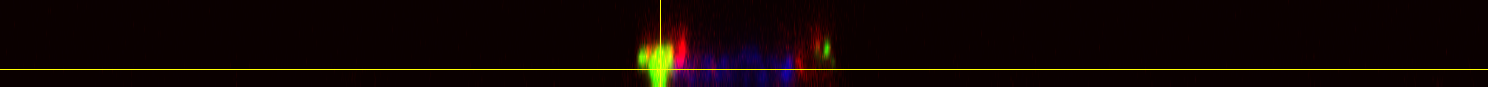

Supplement: Supplementary file 6 — Source Data [file 41467_2025_60271_MOESM6_ESM.zip › SOURCE_DATA/Confocal_microscopy_images_originals_figure_5/Z-stack_M.stadtmanae_Fig.5e/XZ 837-2.tif]

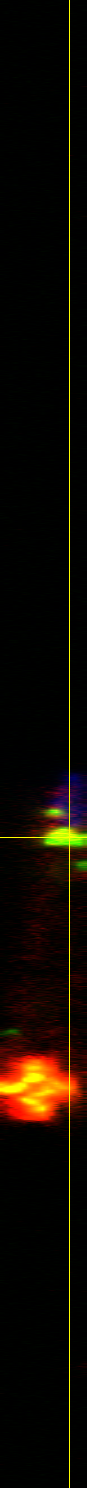

Supplement: Supplementary file 6 — Source Data [file 41467_2025_60271_MOESM6_ESM.zip › SOURCE_DATA/Confocal_microscopy_images_originals_figure_5/Z-stack_M.stadtmanae_Fig.5e/YZ 660-2.tif]

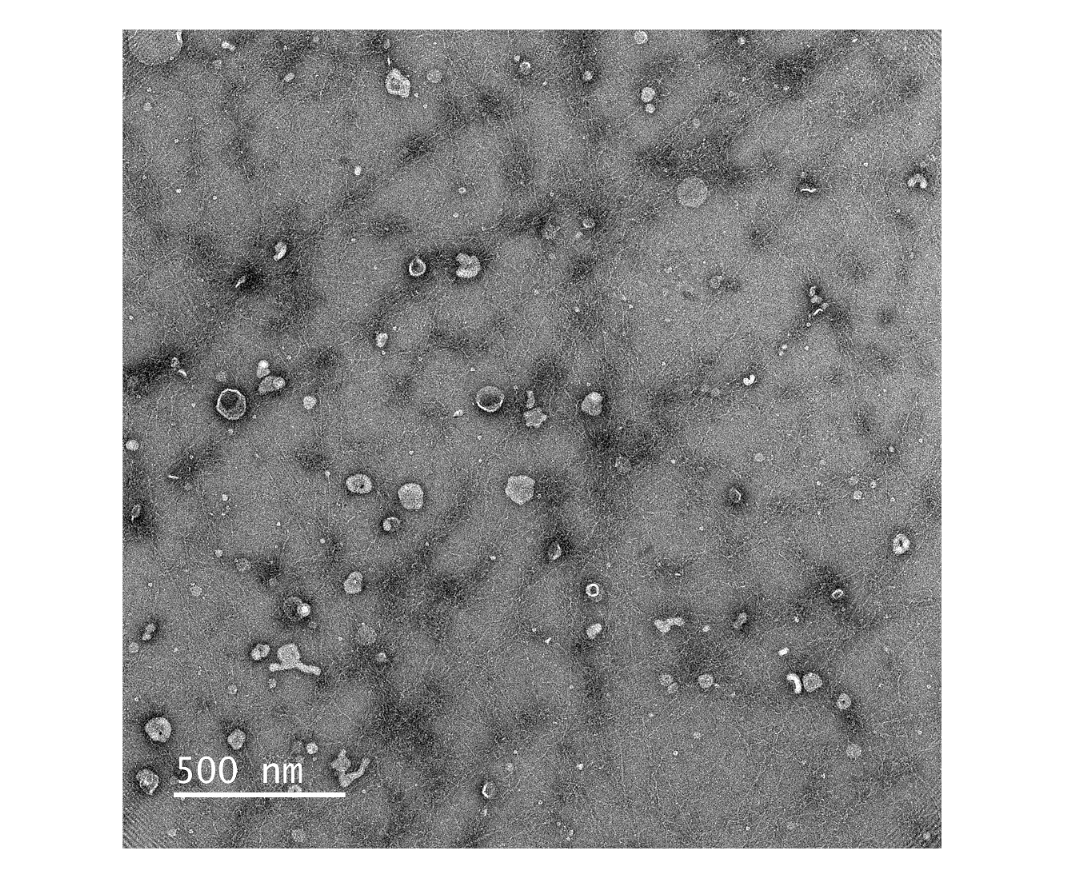

Supplement: Supplementary file 6 — Source Data [file 41467_2025_60271_MOESM6_ESM.zip › SOURCE_DATA/Microscopy images_originals_figure_1/M.smithii_ALI_1d.png]
